# Lactic-fermented egg white reduced serum cholesterol concentrations in mildly hypercholesterolemic Japanese men: a double-blind, parallel-arm design

**DOI:** 10.1186/s12944-017-0499-1

**Published:** 2017-05-30

**Authors:** Ryosuke Matsuoka, Mika Usuda, Yasunobu Masuda, Masaaki Kunou, Kazunori Utsunomiya

**Affiliations:** 1 R&D Division, Kewpie Corporation, 2-5-7 Sengawa Kewport, Sengawa-Cho, Chofu-Shi, Tokyo 182-0002 Japan; 2 Department of Internal Medicine, The Jikei University School of Medicine, 3-25-8, Nishi-shinbashi, Minato-ku, Tokyo 105-8461 Japan

**Keywords:** Egg white protein, Total cholesterol LDL-cholesterol, Hypercholesteroremia, Fermented, Clinical trial

## Abstract

**Background:**

Lactic-fermented egg white (LE), produced by lactic acid fermentation of egg white, is an easy-to-consume form of egg white. Here we assessed the effect of daily consumption of LE for 8 weeks on serum total cholesterol (TC) levels.

**Methods:**

The study followed a double-blind, parallel-arm design and included 88 adult men with mild hypercholesterolemia (mean ± standard error) serum TC levels, 229 ± 1.6 mg/dL; range, 204–259 mg/dL). The subjects were randomly divided into three groups, which consumed LE containing 4, 6, or 8 g of protein daily for 8 weeks. Blood samples were collected before starting LE consumption (baseline) and at 4 and 8 weeks to measure serum TC and low-density lipoprotein cholesterol (LDL-C) levels.

**Results:**

After 8 weeks of consumption, serum TC levels in the 8 g group decreased by 11.0 ± 3.7 mg/dL, a significant decrease compared to baseline (*p* < 0.05) and a significantly greater decrease than for the 4 g group (3.1 ± 3.4 mg/dL; *p* < 0.05). Serum LDL-C levels in the 8 g group decreased by 13.7 ± 3.1 mg/dL, again a significant decrease compared with baseline (*p* < 0.05) and a significantly greater decrease than that for the 4 g group (2.1 ± 2.9 mg/dL; *p* < 0.05).

**Conclusion:**

Consumption of LE for 8 weeks at a daily dose of 8 g of proteins reduced serum TC and LDL-C levels in men with mild hypercholesterolemia, suggesting this may be effective in helping to prevent arteriosclerotic diseases.

**Trial registration:**

This clinical trial was retrospectively registered with the Japan Medical Association Center for Clinical Trials, (JMA-IIA00279; registered on 13/03/2017; https://dbcentre3.jmacct.med.or.jp/JMACTR/App/JMACTRE02_04/JMACTRE02_04.aspx?kbn=3&seqno=6530).

## Background

The prevention of arteriosclerosis is important because its progression can result in serious conditions such as coronary heart diseases and cerebrovascular diseases. A major risk factor for arteriosclerosis is hypercholesterolemia, which has been reported in approximately 30% of the adults in Japan [1]. Because eggs are known to contain cholesterol and their consumption results in an elevation of serum cholesterol levels, hypercholesterolemia patients undergoing dietary therapy are advised to refrain from consuming them [2]. However, many studies have indicated that blood cholesterol levels remain unaffected when healthy individuals consume whole eggs [3–7]. The effect of egg consumption on blood cholesterol levels appears to depend on an individual’s constitutional predisposition, the foods consumed along with the eggs, and the functional constituents of the eggs. A likely relevant predisposition is the individual’s responsiveness to dietary cholesterol: some individuals are prone to increased blood cholesterol in response to dietary cholesterol, whereas blood cholesterol levels have been known not to increase in those less responsive [8–10]. As an example of the effect that the foods consumed with eggs can have, serum cholesterol levels have been reported to increase to a greater extent when eggs are consumed with butter than when consumed with margarine [11, 12].

As an example of the effect of the functional constituents of eggs, phosphatidylcholine in egg yolk has been shown to lower serum cholesterol levels [13]. Egg white contains only a low level of cholesterol; it also contains proteins that have been reported to reduce cholesterol levels. Yamamoto et al. [14] reported that, in rats, serum total cholesterol (TC) levels were significantly lower after consuming isolated soybean proteins and egg white, and high-density lipoprotein cholesterol (HDL-C) levels were significantly higher after egg white consumption, when compared with the respective values after casein consumption. Asato et al. [15] conducted a study in which female university students with slightly high cholesterol levels consumed 23 g as protein of either cheese, tofu or egg white proteins over 30 days. Compared with cheese, egg white proteins significantly reduced serum TC and low-density lipoprotein cholesterol (LDL-C) levels to an extent comparable to that of soybean proteins (Tofu), with their well-characterized cholesterol-lowering effect.

However, egg white has proved difficult to use for dietary therapy because the continued consumption of a large quantity is not easy due to its hydrogen sulfide odor. To resolve this, we developed lactic-fermented egg white (LE), produced through the fermentation of egg white with lactic acid [16]. LE is an easier form of egg white to consume.

It has been reported that the daily consumption of 23 g of egg white proteins had a cholesterol-lowering effect equivalent to that of soybean proteins [15]. However, soybean proteins exert their cholesterol-lowering effect at a daily dose of 6–10 g [17, 18], so it may be possible to reduce blood cholesterol with egg white proteins at a daily dose <23 g. In this study, therefore, we investigated the effects of the consumption of LE in daily doses of 4, 6, and 8 g of proteins on serum TC levels in Japanese men with mild hypercholesterolemia.

## Methods

### Test food

The LE was prepared at the R&D Division, Kewpie Corporation (Tokyo, Japan) [16]. The test food was an easy-to-drink preparation containing sugar, pectin, flavoring agents, and other ingredients in addition to LE containing 4.2, 6.4, or 8.2 g of proteins per serving (the ovalbumin content was 4.27 g per 8.2 g protein). The analyzed nutritional composition per serving (200 g) of the LE preparations is shown in Table 1.

**Table 1:** Nutrient composition of LE drinks

		8 g	6 g	4 g
Energy	(kcal)	124	106	84
Moisture	(g)	169	173	178
Protein	(g)	8.2	6.4	4.2
Fat	(g)	<0.1	<0.1	<0.1
Carbohydrate	(g)	22.6	20.2	17.0
Sodium	(mg)	153	121	84

Analytical Value (per 200 g drink)

### Subjects

The subjects were selected from men registered with SOUKEN Co., Ltd. (Tokyo, Japan). Recruitment was a two-stage process. In the first stage, candidates competed a self-administered questionnaire to ensure they fulfilled the following inclusion criteria: Japanese men with mild hypercholesterolemia, aged 20–65 years, who were not undergoing treatment for hyperlipidemia or diabetes, who had no subjective symptoms of gout, and who were capable of filling out the required forms, such as self-diagnosis forms, and of visiting a designated institution as scheduled. The exclusion criteria were as follows: taking drugs that could potentially affect the test results (e.g., anti-hyperlipidemic agents, anti-diabetic agents, oral corticosteroid formulations, and antihypertensive agents); regular consumption of foods for specified health uses that could potentially affect test results; excessive alcohol consumption; suspected allergic reactions (particularly to egg and milk); participation in other clinical studies; a history of serious liver damage, kidney damage, or myocardial infarction; a history of, or current, hepatitis; and serious anemia. The candidates who fulfilled these criteria then underwent a screening survey including a blood test, with the following further inclusion criteria: serum TC of 200–260 mg/dL; triglycerides (TG) within 2-fold of the upper limit of the reference value; fasting blood glucose ≤110 mg/dL; AST, ALT, and γ-GT levels within 1.5-fold of the upper limit of the respective reference values (as an indication of liver function); creatinine level (as an indicator of kidney function) not exceeding the upper limit of the reference value (1.5 mg/dL); and uric acid ≤9.0 mg/dL). In total, 93 candidates fulfilled the inclusion and exclusion criteria and were considered by the Principal Investigator to be suitable for participation in this study.

When a test result obtained during the course of the study was inconsistent with a result reported by a subject in the initial questionnaire, the subject was still assumed to be eligible to participate unless the item was tested in the screening survey. However, subjects shown by their dietary intake records to have poor dietary habits were considered for exclusion from the analysis set.

This study was conducted as an investigator-initiated clinical study in accordance with the principles of the Declaration of Helsinki, following approval from the SOKEN Human Ethics Committee (authorization number: KYP2–2006-0320). Prior to participation, the objective, methods, and predicted disadvantages of this study were explained to the subjects in detail. All voluntarily elected to participate study and indicated their consent in writing.

### Test protocol

The study followed a double-blind, parallel-arm design. The subjects were divided into three groups (4 g, 6 g, and 8 g groups), minimizing between-group differences in age and in serum TC, low-density lipoprotein cholesterol (LDL-C), and Triglyceride (TG) levels.

According to their group, subjects consumed an LE preparation containing 4, 6, or 8 g of egg white proteins (solid conversion) after their evening meal every day for 8 weeks. Fasting blood samples for the blood analysis were collected early in the morning on 3 days: before starting the LE consumption protocol and 4 and 8 weeks after the start. Subjects were instructed to fast after 22:00 on the day before the blood collection.

### Dietary and physical activity surveys

Subjects recorded what they ate during the day before each of the three blood collections to allow their intake levels of energy, proteins, lipids, carbohydrates, dietary fibers, and cholesterol to be calculated using the nutrition calculation software Excel Eiyoukun ver. 3 (Kenpakusha, Tokyo, Japan). In addition, each subject completed a physical activity survey using a pedometer on those same 3 days.

### Blood tests

The blood analyses were performed by SRL Inc. (Tokyo Japan). Red blood cell and platelet counts were measured by the sheath flow electrical resistance method; white blood cell count by flow cytometry using a semiconductor laser; mean corpuscular volume, mean corpuscular hemoglobin, and mean corpuscular hemoglobin concentration by calculation; hematocrit (Ht) by the red blood cell pulse height detection method; and hemoglobin (Hb) by the SLS-Hb method. Total protein was measured by the Biuret method; albumin by nephelometry; AST, ALT, and γ-GT by a method compatible with the Japan Society of Clinical Chemistry standard; blood urea nitrogen (BUN) by the urease-LED-UV method; Creatinine and Ureic acid by an enzymatic method; blood glucose by the hexokinase ultraviolet method; TC, TG, and free fatty acid (FFA) levels by an enzymatic method; LDL-C and HDL-C by a direct method; sodium (Na), potassium, and chloride (Cl) by an electrode method; and calcium (Ca) by the Arsenazo III method.

### Physical characteristics

The height, weight, body fat percentage, body mass index, muscle mass, and fat mass of each subject were measured before and at 4 and 8 weeks after starting LE consumption, using InBody3.2 (Biospace Co. Ltd., Tokyo, Japan).

### Statistical analysis

Test results are presented as mean ± standard error and differences were considered statistically significant when the significance level was less than 5%. Dunnett’s test was used to assess temporal changes and Tukey-Kramer test was employed for comparisons between the groups. A statistical analysis software, IBM SPSS Statistics 20 (IBM Japan, Ltd., Tokyo) was used for statistical analyses.

## Results

### Subjects

**Fig. 1:**
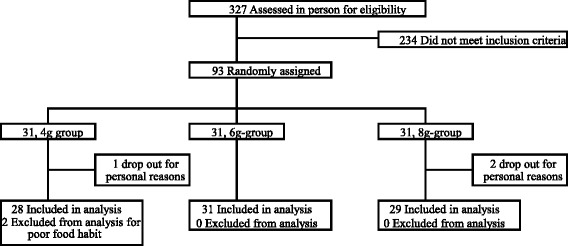
Flowchart of subject recruitment and assignment to the study groups

Figure 1 shows a flowchart of the recruitment. Although this study involved 93 subjects initially, three withdrew for personal reasons and two were determined to be treated as drop-out cases due to poor dietary habits in a case conference. Finally, the analysis set comprised 88 subjects (mean age ± standard error, 42.7 ± 9.1 years; range, 29–63 years) with serum TC levels ≥200 mg/dL (229 ± 1.6 mg/dL; range, 204–259 mg/dL) and who had not been treated. Table 2 presents the background characteristics of the subjects.

**Table 2:** Background of the subjects

	8 g	6 g	4 g
Ages (y)	41.6 ± 1.7	42.8 ± 1.5	43.6 ± 1.9
Height (cm)	170 ± 1	170 ± 1	171 ± 2
Body weight (kg)	70.2 ± 1.7	71.3 ± 2.1	71.9 ± 1.9
BMI (kg/m2)	24.3 ± 0.5	24.8 ± 0.7	24.6 ± 0.6
Total cholesterol (mg/dL)	231 ± 15	228 ± 15	227 ± 15
LDL-cholesterol (mg/dL)	148 ± 12	149 ± 16	146 ± 15
HDL-cholesterol (mg/dL)	58.8 ± 2.5	54.9 ± 2.1	59.1 ± 2.5
Triglyceride (mg/dL)	109 ± 8	116 ± 8	109 ± 8
Systric blood presure (mmHg)	122 ± 16	118 ± 15	115 ± 12
Diastric blood pressure (mmHg)	78.2 ± 11.7	75.8 ± 11.8	75.1 ± 10.0

Mean ± SE of 29 (8 g), 31 (6 g), and 28 (4 g)

### Dietary and physical activity surveys

Table 3 presents the subjects’ dietary intake of energy and nutrients during the study period. The dietary fiber intake was significantly lower after 4 and 8 weeks in the 8 g group compared with the level prior to the consumption of LE (baseline) but was not significantly different in the 4 g and 6 g groups. For the other nutrients, there were no significant differences compared with baseline in any group and no significant differences between the groups. In the pedometer-based physical activity survey, no group showed a significant change from baseline, and no significant intergroup difference was noted. At 8 weeks, the activity levels were as follows: 4 g group, 9190 ± 3797 steps/day; 6 g group, 8584 ± 4251 steps/day; and 8 g group, 10,063 ± 4077 steps/day.

**Table 3:** Dietary intake of subjects

			Intake Period (Weeks)		
	Groups	n	0	4	8
Energy (kcal)	8 g	29	2203 ± 349	2156 ± 334	2205 ± 407
	6 g	31	2159 ± 436	2058 ± 415	2143 ± 505
	4 g	28	2238 ± 440	2214 ± 397	2290 ± 387
Protein (g)	8 g	29	76.2 ± 16.0	74.8 ± 15.4	75.3 ± 18.0
	6 g	31	75.9 ± 21.6	73.5 ± 20.2	77.3 ± 20.5
	4 g	28	75.3 ± 16.2	75.9 ± 16.5	77.3 ± 15.0
Fat (g)	8 g	29	67.7 ± 19.0	63.9 ± 17.9	66.6 ± 15.1
	6 g	31	65.3 ± 22.1	60.4 ± 16.2	65.3 ± 18.1
	4 g	28	66.4 ± 18.5	68.4 ± 18.7	71.2 ± 16.5
Carbohydrate (g)	8 g	29	290 ± 59	281 ± 55	285 ± 65
	6 g	31	285 ± 61	272 ± 69	275 ± 65
	4 g	28	288 ± 65	294 ± 51	299 ± 61
Cholesterol (mg)	8 g	29	330 ± 152	319 ± 134	307 ± 132
	6 g	31	370 ± 173	303 ± 142	358 ± 151
	4 g	28	316 ± 125	333 ± 144	341 ± 161
Dietary fiber (g)	8 g	29	13.2 ± 3.4	11.4 ± 2.7*	11.5 ± 3.3*
	6 g	31	12.4 ± 4.2	11.1 ± 3.6	11.4 ± 3.7
	4 g	28	12.8 ± 3.3	11.5 ± 3.1	12.7 ± 3.7

Mean ± SE of 29 (8 g), 31 (6 g), and 28 (4 g), *: *p* < 0.05 vs. 0 weeks by Dunnett test

### Changes in TC, LDL-C, HDL-C and TG levels

**Fig. 2:**
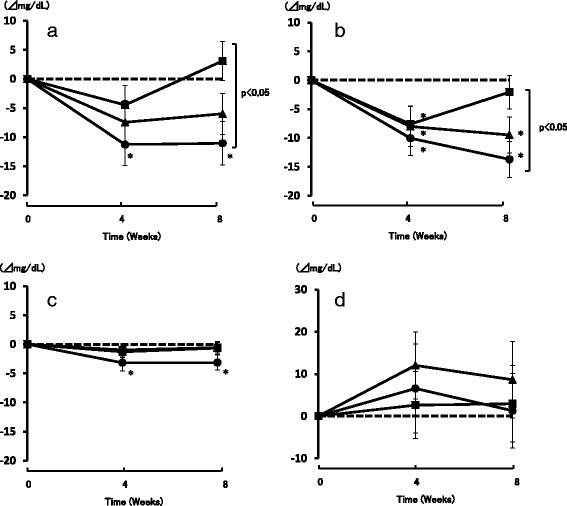
Change in serum total cholesterol (**a**), LDL-cholesterol (**b**), HDL-cholesterol (**c**), and triglyceride (**d**) levels in subjects who consumed preparations containing 4 g, 6 g, or 8 g of lactic-fermented egg white protein for 8 weeks. ■ 4 g group (*n* = 29) ▲ 6 g group (*n* = 31) ● 8 g group (*n* = 28). The values are mean ± SE

Changes in TC from baseline (ΔTC) were calculated. Significant decreases were observed after 4 and 8 weeks in the 8 g group (−11.3 mg/dL, *p* < 0.01 and −11.0 mg/dL, *p* < 0.05, respectively), and a near-significant decrease was observed after 4 weeks in the 6 g group (*p* = 0.077). Furthermore, ΔTC at 8 weeks was significantly greater in the 8 g group than in the 4 g group (*p* < 0.05; Fig. 2a).

Significant decreases from baseline in serum LDL-C levels were observed after 4 and 8 weeks in the 8 g group (−10.0 mg/dL, *p* < 0.01 and −13.7 mg/dL, *p* < 0.001, respectively), after 4 and 8 weeks in the 6 g group (−8.0 mg/dL, *p* < 0.05 and −9.5 mg/dL, *p* < 0.01, respectively), and after 4 weeks in the 4 g group (−7.6 mg/dL, *p* < 0.05). Serum LDL-C levels at 8 weeks were significantly lower in the 8 g group than in the 4 g group (*p* < 0.05; Fig. 2b).

In the 8 g group, the serum HDL-C levels were significantly decreased from baseline after 4 and 8 weeks (*p* < 0.05; Fig. 2c), but there was no significant change in the ratio of LDL-C and HDL-C levels (LDL-C/HDL-C) (Table 4). However, significant decreases in LDL-C/HDL-C were observed in the 6 g group after 8 weeks and in the 4 g group after 4 and 8 weeks (Table 5). Serum TG levels did not change significantly over the study period in any group (Fig. 2d).

**Table 4:** Serum Biochemical Analysis

			Intake Period (Weeks)		
	Groups	n	0	4	8
NEFA (mEq/L)	8 g	29	0.517 ± 0.035	0.467 ± 0.029	0.376 ± 0.028*
	6 g	31	0.512 ± 0.034	0.489 ± 0.036	0.419 ± 0.034*
	4 g	28	0.511 ± 0.044	0.441 ± 0.041	0.414 ± 0.042*
LDL-C/HDL-C	8 g	29	2.65 ± 0.11	2.60 ± 0.12	2.51 ± 0.11
	6 g	31	2.86 ± 0.14	2.75 ± 0.13	2.72 ± 0.14*
	4 g	28	2.60 ± 0.12	2.50 ± 0.12*	2.58 ± 0.12*
Total protein (mg/dL)	8 g	29	7.28 ± 0.07	7.23 ± 0.07	7.22 ± 0.06
	6 g	31	7.24 ± 0.07	7.19 ± 0.06	7.27 ± 0.07
	4 g	28	7.29 ± 0.07	7.27 ± 0.07	7.31 ± 0.07
Albumin (mg/dL)	8 g	29	4.63 ± 0.03	4.67 ± 0.03	4.54 ± 0.03*
	6 g	31	4.54 ± 0.03	4.89 ± 0.04	4.54 ± 0.03
	4 g	28	4.55 ± 0.03	4.41 ± 0.04	4.53 ± 0.04
Glucose (mg/dL)	8 g	29	92.9 ± 1.2	93.7 ± 1.7	92.9 ± 1.4
	6 g	31	90.4 ± 1.1	92.2 ± 0.9	91.1 ± 0.9
	4 g	28	92.8 ± 1.6	92.9 ± 1.3	93.8 ± 1.4

Mean ± SE of 29 (8 g), 31 (67 6 g), and 28 (4 g), *: *p* < 0.05 vs. 0 weeks by Dunnett test

**Table 5:** Results of hematological tests

			Intake Period (Weeks)		
	Groups	n	0	4	8
WBC (/μL)	8 g	29	5852 ± 258	5731 ± 198	5586 ± 264
	6 g	31	5877 ± 240	5755 ± 266	5926 ± 299
	4 g	28	6336 ± 316	6004 ± 257	5979 ± 284
RBC (/μL)	8 g	29	498 ± 5	489 ± 6	495 ± 5
	6 g	31	494 ± 7	485 ± 6*	488 ± 7
	4 g	28	489 ± 5	489 ± 4	490 ± 5
Hb (g/dL)	8 g	29	15.1 ± 0.2	14.9 ± 0.2	15.0 ± 0.2
	6 g	31	15.0 ± 0.2	14.7 ± 0.2*	14.8 ± 0.2
	4 g	28	14.7 ± 0.1	14.8 ± 0.1	14.8 ± 0.1
Ht (%)	8 g	29	46.6 ± 0.5	45.7 ± 0.5	46.7 ± 0.5
	6 g	31	46.4 ± 0.5	45.3 ± 0.5*	45.8 ± 0.6
	4 g	28	45.8 ± 0.4	45.6 ± 0.4	45.9 ± 0.4
MCV (fl)	8 g	29	93.6 ± 0.6	93.5 ± 0.6	94.2 ± 0.6
	6 g	31	94.1 ± 0.6	93.5 ± 0.6	94.0 ± 0.6
	4 g	28	93.6 ± 0.6	93.2 ± 0.6	93.7 ± 0.6
MCH (pg)	8 g	29	30.3 ± 0.2	30.4 ± 0.2	30.4 ± 0.2
	6 g	31	30.4 ± 0.2	30.3 ± 0.2	30.3 ± 0.2
	4 g	28	30.1 ± 0.2	30.2 ± 0.2	30.2 ± 0.2
MCHC (%)	8 g	29	32.4 ± 0.1	32.6 ± 0.1	32.2 ± 0.1
	6 g	31	32.2 ± 0.1	32.4 ± 0.1	32.2 ± 0.1
	4 g	28	32.2 ± 0.1	32.4 ± 0.1	32.3 ± 0.1
Platelet (/μL)	8 g	29	25.3 ± 0.8	25.0 ± 1.0	25.0 ± 0.9
	6 g	31	25.7 ± 1.3	24.8 ± 1.2	25.2 ± 1.2
	4 g	28	24.5 ± 0.9	24.2 ± 0.9	24.4 ± 0.8

Mean ± SE of 29 (8 g), 31 (6 g), and 28 (4 g), *: *p* < 0.05 vs. 0 weeks by Dunnett test

### Other blood test findings

Significant decreases in serum FFA levels were observed after 8 weeks in all three groups, but there were no significant intergroup differences (Table 4). The red blood cell count and Hb and Ht levels decreased significantly in the 6 g group after 4 weeks.

In the 8 g group, albumin levels decreased significantly after 8 weeks, BUN levels increased significantly after 4 weeks, Na and Cl levels increased significantly after 8 weeks, and Ca levels decreased significantly after 8 weeks. Although these changes were statistically significant, the values remained within the reference ranges and represented no clinical concerns (Tables 5 and 6).

**Table 6:** Hepatic and kidney condition and serum mineral levels

			Intake Period (Weeks)		
	Groups	n	0	4	8
AST (IU/L)	8 g	29	20.1 ± 0.9	20.3 ± 0.8	20.8 ± 0.8
	6 g	31	20.3 ± 1.0	20.8 ± 1.0	20.9 ± 1.2
	4 g	28	22.9 ± 1.5	21.8 ± 1.1	21.8 ± 1.1
ALT (IU/L)	8 g	29	24.2 ± 1.8	23.7 ± 1.7	24.1 ± 1.7
	6 g	31	25.6 ± 2.0	24.8 ± 2.3	24.7 ± 2.0
	4 g	28	27.6 ± 2.3	25.3 ± 2.4	27.4 ± 2.6
γ-GTP (IU/L)	8 g	29	40.7 ± 4.6	40.4 ± 4.8	42.6 ± 4.9
	6 g	31	37.9 ± 4.0	35.8 ± 3.4	35.7 ± 3.9
	4 g	28	39.1 ± 3.6	40.0 ± 4.1	42.1 ± 5.1
BUN (mg/dL)	8 g	29	12.4 ± 0.5	13.7 ± 0.5*	13.2 ± 0.5
	6 g	31	13.8 ± 0.6	14.9 ± 0.7	14.6 ± 0.6
	4 g	28	13.4 ± 0.7	13.9 ± 0.6	13.3 ± 0.5
Cr (mg/dL)	8 g	29	0.820 ± 0.020	0.822 ± 0.021	0.829 ± 0.023
	6 g	31	0.791 ± 0.015	0.795 ± 0.016	0.791 ± 0.015
	4 g	28	0.814 ± 0.016	0.811 ± 0.015	0.811 ± 0.013
UA (mg/dL)	8 g	29	6.09 ± 0.18	6.03 ± 0.17	6.10 ± 0.21
	6 g	31	6.15 ± 0.17	6.11 ± 0.18	6.02 ± 0.15
	4 g	28	6.02 ± 0.22	6.00 ± 0.23	6.07 ± 0.24
Na (mg/dL)	8 g	29	142 ± 0	142 ± 0	142 ± 0
	6 g	31	142 ± 0	142 ± 0	142 ± 0
	4 g	28	142 ± 0	142 ± 0	142 ± 0*
K (mg/dL)	8 g	29	4.10 ± 0.05	4.11 ± 0.04	4.20 ± 0.05
	6 g	31	4.16 ± 0.05	4.07 ± 0.04	4.22 ± 0.04
	4 g	28	4.24 ± 0.06	4.16 ± 0.05	4.19 ± 0.04
Cl (mg/dL)	8 g	29	103 ± 0	103 ± 0	104 ± 0
	6 g	31	103 ± 0	103 ± 0	104 ± 0
	4 g	28	103 ± 0	103 ± 0	104 ± 0*
Ca (mg/dL)	8 g	29	9.43 ± 0.05	9.33 ± 0.07	9.24 ± 0.06*
	6 g	31	9.32 ± 0.05	9.24 ± 0.06	9.26 ± 0.04
	4 g	28	9.43 ± 0.05	9.34 ± 0.06	9.34 ± 0.06

Mean ± SE of 29 (8 g), 31 (6 g), and 28 (4 g), *: *p* < 0.05 vs. 0 weeks by Dunnett test

### Safety

No adverse events were observed in any subject on the basis of subjective symptoms or interviews by physicians before and after the 8-week period (data not shown).

## Discussion

In this study, we evaluated the effects of LE on serum TC and LDL-C levels. Although consumption of 23 g of egg white proteins has previously been shown to reduce blood cholesterol levels, the present study showed that consumption of LE in a dose equivalent to 6 g of proteins resulted in a significant decrease in LDL-C levels, and that 8-g consumption resulted in significant decreases in serum LDL-C and TC levels.

Serum cholesterol-lowering effects in humans have been reported for soybean proteins and other plant-derived proteins [17, 18]. Consumption of 7 g of soybean proteins daily for 12 weeks has been reported to reduce blood TC levels by 5.6% (from 245.9 mg/dL to 232.1 mg/dL) and blood LDL-C levels by 8.6% (from 159.2 mg/dL to 145.5 mg/dL) [17]. In the present study, LE consumption for 8 weeks at a daily dose equivalent to 8 g of egg white proteins significantly reduced serum TC levels by 4.8% (from 230.8 mg/dL to 219.7 mg/dL) and serum LDL-C levels by 9.2% (from 148.3 mg/dL to 134.6 mg/dL). These results indicate that 7 g of soybean proteins and 8 g of egg white proteins are nearly equivalent in their physiological activity. This is in agreement with a report from Yamamoto et al. stating that soybean and egg white make equivalent contributions to blood cholesterol levels [16].

As well as proteins, other food-related components are also known to have a serum cholesterol-lowering effect, including dietary fiber [19, 20], plant sterols [21], chitosan [22, 23], low-molecular-weight Na alginate [24], and tea catechins [25]. The percentage reductions in serum TC and LDL-C reported for these materials are 5.9 and 5.7% for dietary fiber [20], 5.7 and 11.1% for plant sterols [26], and 3.8 and 5.3% for tea catechins [25]. Thus, the effects of the egg white proteins in reducing serum TC and LCL-C were comparable in percentage to those of the other reported food-related components.

Several different mechanisms are known to underlie the cholesterol-lowering action of functional food components, including the inhibition of cholesterol dissolution into bile acid micelles reported for plant sterols [27], the acceleration of bile acid excretion by soybean proteins and chitosan [28], a combination of these two mechanisms reported for low-molecular-weight Na alginate [29], and cholesterol desorption from bile acid micelles for tea catechins [30].

In general, the consumption of food that inhibits cholesterol absorption is known to lower serum LDL-C levels, and plant sterols have also been reported to reduce serum LDL-C levels [26]. A decrease in LDL-C levels after egg white consumption was also observed in the present study, suggesting that the egg white may have inhibited the absorption of cholesterol. In experiments on thoracic lymph duct-cannulated rats, egg white proteins were reported to have significantly suppressed cholesterol absorption into lymph in comparison with casein; ovalbumin and ovotransferrin in egg white were reported as an underlying mechanism that inhibited the dissolution of cholesterol into bile acid micelles [31, 32]. In addition, egg white ovomucin has been reported as inhibiting cholesterol uptake in the intestine model cell line Caco-2 [33].

Other possible mechanisms suggested for the cholesterol-lowering effect of egg white include the suppression of VLDV release from the liver and acceleration of cholesterol catabolism in the liver by a sulfur-containing amino acid (cystine), which is abundantly found in egg white [34]. However, uncertainty regarding these mechanisms has been reported and the details remain to be studied [31].

In addition to the intrinsic effects of egg white, the lactic fermentation process may have contributed to the effects observed. The LE used in the present study was prepared by fermenting egg white using lactobacilli. A mixture of *Streptococcus thermophilus* and *Lactobacillus delbrueckii* subsp. *bulgaricus*, which are commonly used in yogurt preparation, was used for the fermentation in this study. Lactic acid-fermentation products have been reported to show cholesterol-lowering effects in humans [35, 36]. However, the bacterial mixture used in the present study did not include the *Lactobacillus* strain reported to have the serum cholesterol-lowering effect and has been reported to show no serum TC- or LDL-C-lowering effects [37]. It is therefore likely that the serum cholesterol-lowering effect observed in the present study was mainly due to the effect of the egg white proteins, which has previously been reported in rats and humans, and that the effect of the lactobacilli was negligible or non-existent [14, 15].

High serum TC and LDL-C levels and low serum HDL-C levels are risk factors for arteriosclerosis [38, 39]. In our 8 g group, the serum TC and LDL-C levels were significantly reduced, but the serum HDL-C levels also decreased significantly. Nevertheless, LDL-C/HDL-C did not change in the 8 g group, and the overall effect of LE consumption, therefore, was considered to have driven the subjects’ serum cholesterol profiles toward the anti-arteriosclerotic direction.

A stratified analysis dividing the subjects into those with a slightly high initial serum TC level (≥220 mg/dL) and those with initial TC <220 mg/dL revealed that the serum TC level significantly decreased in the former group, whereas no significant change was found in the latter group (data not shown). A similar phenomenon is commonly observed with functional foods, such as plant sterols, that lower serum cholesterol levels, confirming that LE consumption did not reduce serum TC and LDL-C levels when taken by healthy humans.

## Conclusions

These results suggest that consumption of LE for 8 weeks at a daily dose of 8 g of proteins was effective for reducing serum TC and LDL-C levels in individuals with mild hypercholesterolemia. LE consumption may therefore help in the prevention of arteriosclerotic diseases.

## Abbreviations

FFA: Free fatty acid
HDL-C: High-density lipoprotein cholesterol
LDL-C: Low-density lipoprotein cholesterol
LE: Lactic-fermented egg white
TC: Total cholesterol
TG: Triglyceride
